# Overview of myelin, major myelin lipids, and myelin-associated proteins

**DOI:** 10.3389/fchem.2022.1041961

**Published:** 2023-02-21

**Authors:** Alexander Kister, Ilya Kister

**Affiliations:** Department of Neurology, New York University Grossman School of Medicine, New York, NY, United States

**Keywords:** myelin, myelin protein, lipid membrane, myelination, glia

## Abstract

Myelin is a modified cell membrane that forms a multilayer sheath around the axon. It retains the main characteristics of biological membranes, such as lipid bilayer, but differs from them in several important respects. In this review, we focus on aspects of myelin composition that are peculiar to this structure and differentiate it from the more conventional cell membranes, with special attention to its constituent lipid components and several of the most common and important myelin proteins: myelin basic protein, proteolipid protein, and myelin protein zero. We also discuss the many-fold functions of myelin, which include reliable electrical insulation of axons to ensure rapid propagation of nerve impulses, provision of trophic support along the axon and organization of the unmyelinated nodes of Ranvier, as well as the relationship between myelin biology and neurologic disease such as multiple sclerosis. We conclude with a brief history of discovery in the field and outline questions for future research.

## 1 Introduction

**FIGURE 1:**
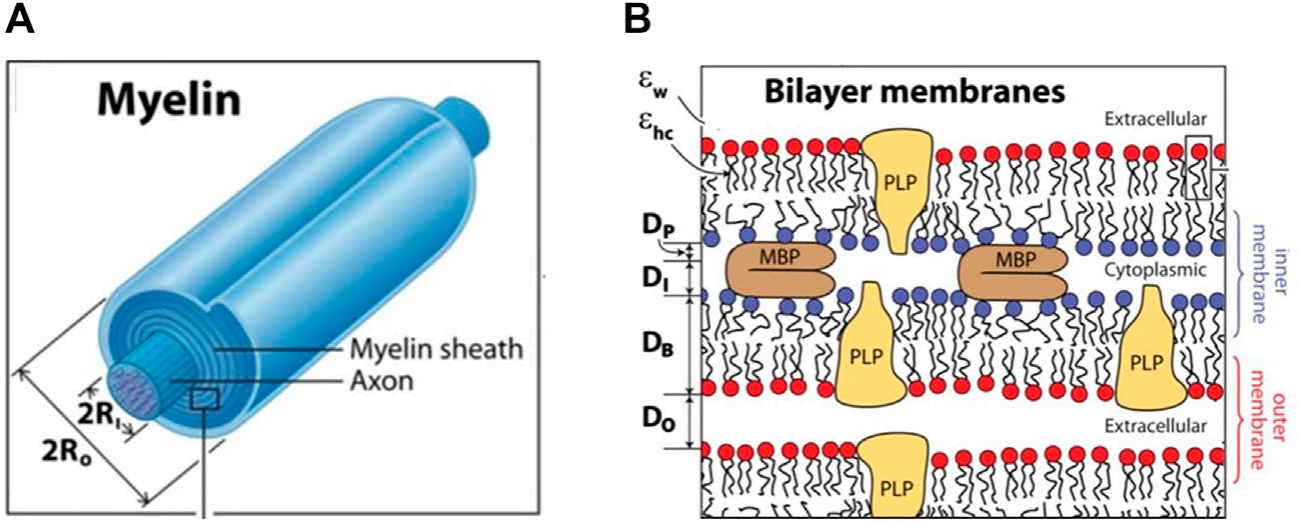
The structure of the myelin sheath. The myelinated axon **(A)**, bilayer membranes **(B)**. **(A)**: The ratio of 2xRo to 2xRi (in A) is the g-ratio. Ri—inner radius; Ro—outer radius **(B)**: Each bilayer of thickness DB is separated by cytoplasmic and extracellular water gaps of thicknesses DI and DO, and effective protein thickness DP is occupied by the fraction of MBP constituting the cytoplasmic water gap. Adapted from Min Y, et al. Proc Natl Acad Sci U S A. 106(9): 3154–3159.

Myelin sheath is a modified cell membrane that wraps multiple times around the nerve axon (Figure 1). Tight, layer-by-layer packing allows for reliable electrical insulation of axons and thereby ensures rapid propagation of nerve impulses—electromagnetic waves driven by electric potential - along the axon and reduce axonal energy consumption. Compact multilayered myelin sheath allows an increase in the velocity of propagation from less than 1 m/s to 50–100 m/s without an increase in the diameter of axons. Myelin sheath is an exclusive innovation of vertebrate organisms and may explain the larger size of vertebrates relative to nearly all other animals (Zalc, 2006).

Optimum insulation depends on the types and ratios of myelin constituent lipids and proteins and myelin water fraction. If the myelin sheath is damaged, axonal insulation is disrupted, and nerve impulses along the axon slow down or fail to conduct, resulting in neurologic dysfunction. Myelin-related pathology underlies several neurogenetic diseases, such as leukodystrophies and inherited demyelinating neuropathies, and acquired neurologic diseases, such as multiple sclerosis (MS) and subacute combined degeneration (Harayama & Riezman, 2018). Myelin degradation also contributes to age-related cognitive decline (Bonetto et al., 2021). It is, therefore, important to understand at the molecular level the processes that underlie the formation of the myelin sheath (myelination) and the replacement of damaged areas of the sheath (remyelination).

In this review, we will discuss the general properties of myelin, focusing on the features of its composition, formation, structure, and function that differentiate it from the more conventional cell membranes. We will also address differences in myelin formation and properties in the central nervous system (CNS) and the peripheral nervous system (PNS).

## 2 Myelin sheath and the g-ratio

The myelin sheath is typically made of up to 100 layers tightly wound on top of each other around the axon (Figure 1A) (Simons and Nave, 2015). Two characteristic periodic morphological features of the myelin sheath are alternating major dense lines and intraperiod lines. The major dense lines are ∼two to three nm wide and are formed by the closely condensed intracellular (cytoplasmic) surfaces between the inner membranes of the two lipid bilayers, as shown in Figure 1B. The intraperiod lines are wider—4 nm - and are formed by tightly apposed extracellular surfaces of myelin sheaths.

The number of myelin layers determines the thickness of the sheath, which depends on the axon diameter: the larger the axon, the thicker the myelin sheath. The relative thickness of a myelin sheath is conventionally measured as the ratio between the inner diameter and the outer diameter of the myelin sheath–so-called the g-ratio–as shown in Figure 1A. Thus, the thinner the myelin sheath, the closer the g-value is to 1. The optimal g-ratio depends on the requirement to optimize conduction speed and minimize conduction delays, as well as other properties of the system as a whole, such as the need to conserve volume, especially within the intracranial space. The optimal g-ratio was estimated to be ∼0.77 for CNS and ∼0.6 for PNS (Chomiak and Hu, 2009). Deviations from the optimal g-ratio may result in abnormal neural development and neurologic disease (York et al., 2021).

Quantitative determination of the g-ratio of myelin is done using electron microscopy; recent developments have made this less time-consuming (Kaiser et al., 2021). It is also possible to estimate g-ratio in the brain *in vivo* using advanced magnetic resonance imaging (MRI) techniques (Stikov et al., 2015; West et al., 2016). In healthy subjects, the g-ratio varies by brain region, with higher myelin content in the highly interconnected ‘hub regions’ than in the peripheral connections (Mancini et al., 2018). In patients with MS, an acquired demyelinating disorder, g-ratio-weighted nodal strength in motor, visual, and limbic regions correlates with disease severity (Kamagata et al., 2019). However, wide application of g-ratio estimation to clinical practice is hindered by the large variability of g-values obtained using various MRI techniques (Ellerbrock, Mohammadi, 2018). Comparisons of five different methods of g-ratio estimation in healthy subjects and multiple sclerosis patients showed high variability of g-values, mostly in MS lesions, and two MRI methods did not correctly predict the degree of demyelination in MS lesions (Berg et al., 2022).

## 3 Glial cells and myelinogenesis in the central and the peripheral nervous system

The nervous system is traditionally divided into CNS and PNS. The CNS is comprised of the brain, spinal cord, olfactory and optic nerves, and is myelinated by oligodendrocytes. The PNS is comprised of nerves outside of the CNS–the remaining ten pairs of cranial nerves, spinal nerve roots, and peripheral nerves, and is myelinated by a different type of glial cell—the Schwann cell. The border between central and peripheral myelin–the so-called Obersteiner-Redlich zone—lies along cranial nerves and spinal nerve roots, within a few mm of nerve root entry into the brainstem or the spinal cord. The part of the axon proximal to the Obersteiner-Redlich zone (nearer the cell body) is myelinated with central myelin made by oligodendrocytes, and the part of the axon distal to this zone (farther from the cell body) is myelinated with peripheral myelin made by Schwann cells.

A single oligodendrocyte myelinates between 40 and 60 different axons but only one segment per axon (Simons and Nave, 2015). Thus, each axon in the CNS is myelinated by multiple oligodendrocytes, and each oligodendrocyte myelinates multiple axons. Oligodendrocytes myelinate different axons to variable extents depending on axon diameter to maintain optimal g-ratio. Thus, the same oligodendrocyte will myelinate the larger axons more extensively, yielding a thicker myelin sheath compared to the smaller axons (Waxman and Sims, 1984). An oligodendrocyte typically needs only about 5 h to generate all its myelin, which includes the synthesis of all the necessary proteins and lipids (Czopka et al., 2013).

Within the PNS, Schwann cell myelinates only a single axon, not multiple axons, as do oligodendrocytes in the CNS. Peripheral axons’ often span considerable length, and many Schwann cells are required to myelinate the length of a single axon. The diameter of axons in the PNS ranges from ∼0.1 μm to ∼20 μm, while in the CNS, the axons tend to be smaller, ranging from <0.1 μm to >10 μm in diameter. (Stassart et al., 2018).

Another important distinction between oligodendrocytes and Schwann cells is that Schwann cells myelinate only axons that are greater than 1 μm in diameter, a process called ‘radial sorting’. The wider-diameter peripheral axons conduct impulses at a higher speed than narrower axons, and myelination of the wider axons allows for a further increase in the speed and distance of conducted signal (Feltri et al., 2016). Another feature of myelin sheath found only in the peripheral nerves is Schmidt-Lanterman incisures (SLI): cytoplasmic channels that pass through myelin and connect to the cytoplasm at the edge of the myelin sheath. SLI are formed where there is no tight interaction of adjacent myelin membranes, i.e., not within compact myelin sheath. SLI has a circular-truncated cone shape and are described as ‘beads in a stretched state’ (Terada et al., 2019).

Although CNS and PNS myelin are formed by different glial cell types, they share similar morphological structures, with some quantitative differences in their lipid composition and more substantial qualitative differences in protein composition. The differences between PNS and CNS myelin may explain why some diseases, such as acute inflammatory demyelinating polyneuropathy, affect only peripheral myelin while others, such as multiple sclerosis—only central myelin. Understanding the differences between the two types of myelin may yield clues into the pathogenesis of these disorders and the processes that underlie myelin degeneration in the nervous system (Quarles, 2005).

Other glial cells—astrocytes and microglia–contribute indirectly to myelinogenesis (Bilimoria and Stevens, 2015; Traiffort et al., 2020). Astrocytes promote the development of myelinating oligodendrocytes and accelerate myelin growth. Microglia remove damaged neurons and promote recovery by eliminating degenerated myelin that accumulates with aging and disease (Prineas et al., 2001; Bsibsi et al., 2014). In early development, myelin with ultrastructural abnormalities is phagocytosed by microglia (Djannatian et al., 2021). Microglia also play a neuroprotective and regenerative role by supporting myelination of axons during development and across the lifespan (Lenz and Nelson, 2018; Santos and Fields, 2021). Interestingly, Schwann cells also participate in myelin clearance after nerve injury (Brosius et al., 2017).

## 4 Diverse functions of myelin

In addition to creating tightly packed multilayered insulating segments called ‘internodes’ around the axon, myelin also plays a role in the assembly of the unmyelinated nodes of Ranvier (NR) between the internodes. The NRs are located roughly equidistant from each other along the axon and are the only points of contact between a myelinated axon and the extracellular environment. The main function of NR is to recharge neuron impulses, ensuring signal spreads along the entire length of the axon, which may be over a meter long in humans. Since the impulse appears to ‘leap’ from one NR to another, this process is known as “saltatory conduction”, from the Latin ‘saltus’ –a leap. The mechanism underlying saltatory conduction relies on clusters of voltage-gated Na+ and K+ channels within NR, which open and close depending on changes in the membrane potential of the NR.

Formation of ion channel cluster in the NR, reviewed in (Rasband and Peles, 2021), involves multiple players: cytoskeletal scaffold proteins actin, ankyrin G, beta IV spectrin (Leterrier C. et al., 2015), adhesion molecule neurofascin (Alpizar et el., 2019) and others. Myelin proteins are also essential in NR formation as they attach the myelin sheath to the axon on both sides of the node and thereby ‘fix’ the size of NR. An increase in NR length may alter conduction speed by ∼20%, similar to the effect produced by altering the number of myelin wraps or the internode length (Arancibia-Cárcamo et al., 2017). Because myelin is necessary for NR assembly and ‘size fixing’, problems with myelination also compromise NR function and thereby further impair saltatory conduction and exacerbate neurologic dysfunction (Arancibia-Carcamo and Attwell, 2014).

By insulating the axon along its length, the myelin sheath also inhibits access to nutrients from the extracellular compartment to the axon. An area of intense interest is whether myelin sheath may also serve for the provision of trophic support to the underlying axon. It has been postulated that oligodendrocytes can switch their own intermediate metabolism so that the end-product of glycolysis is lactate, which is then taken up by an axon and used by axonal mitochondria to generate ATP (Nave et al., 2010). The process of lactate delivery from oligodendrocytes to axon requires the formation of narrow cytosolic channels, such as Schmidt–Lanterman incisures discussed above, that connect the glial cell body with the axon during myelination (Spiegel and Peles, 2002). Such channels may exist in non-compact myelin, which differs from compact myelin in its molecular structure. An oligodendrocyte-specific protein 2′,3′-cyclic nucleotide 3′-phosphodiesterase (CNP) is essential for preserving cytoplasmic spaced between inner leaflets of non-compact myelin (Snaidero et al., 2017), as will be discussed below. It is also possible that oligodendrocytes provide energy supplies to axons *via* exosomes (Frühbeis et al., 2020). Failure of the energy-trophic function of oligodendrocytes may contribute to axonal neurodegeneration (Nave et al., 2010; Tepavčević, 2021).

## 5 An overview of myelin composition

Myelin sheath, like all cell membranes, is constituted of three main components - water, lipids, and protein molecules, but the ratio of these components in myelin differs from the respective ratio of a more typical cell membrane. The dry myelin sheath is characterized by a high proportion of lipids (70%–85%) and a low proportion of proteins (15%–30%), while the typical cell membrane has an approximatively equal ratio of proteins to lipids (50%/50%) (Poitelon et al., 2020). The high proportion of lipids in myelin makes it less permeable to ions and a better electrical insulator. It also affects the membrane’s physical properties, such as rigidity and membrane deformation (Harayama and Riezman, 2018). Myelin is highly susceptible to changes in its composition, and even small changes in the ratio of its constituent elements can result in the breakdown of myelin structure (Chrast et al., 2011).

### 5.1 Myelin water

Quantitative electron microscopy (electron probe X-ray microanalysis) shows that CNS myelin *in situ* is 33%–55% water, the lowest water content of any morphological compartment (LoPachin et al., 1991). Near the polar phospholipid headgroups, water molecules have an electrostatic orienting effect and form bonds with the hydrophilic groups of lipid and myelin proteins. Myelin prevents water diffusion transversally to the axon and thereby contributes to anisotropy. Therefore, an increase in anisotropy reflects an increase in myelination (Almeida and Lyons, 2017). A change in myelin concentration has a profound impact on the signal strength on magnetic resonance imaging (MRI), and loss of signal on certain sequences may be a biomarker for myelin degeneration (Abel et al., 2020; Edwards et al., 2022). Advanced MRI techniques can differentiate water protons interacting with lipid bilayers (lipid-associated) from intra- and extracellular water protons (Watanabe et al., 2019).

### 5.2 Myelin lipids

Lipids differ from other major biological macromolecules in that they do not form polymers *via* covalent bonding of monomers but self-assemble due to the hydrophobic effect into macromolecular aggregates, such as lipid bilayer, the basic structure of all cell membranes. Lipids are the main constituents of membranes, but myelin differs from the typical cell membrane in the overall higher proportions of lipids, as well as in the ratio of three major classes of lipid components. In myelin sheath, the proportion of major lipid components is 40% cholesterol, 40% phospholipids, and 20% glycolipids, while in most biological membranes, the ratio is closer to 25%:65%:10%, respectively (Poitelon et al., 2020). Thus, the relative contribution of cholesterol and glycolipids is greater in the formation of a unique multilayer compact myelin structure than in conventional membranes. Slight changes in lipid composition in myelin can alter the intermembrane adhesive properties and lead to the destruction of the myelin structures (Chrast et al., 2011) and serious neurologic illness (Lamari et al., 2013).

Lipids are not directly genetically encoded, but they are synthesized by genetically-encoded enzymes. Thus, myelinogenesis is a strictly regulated process involving the coordinated expression of genes coding for enzymes involved in myelin lipid synthesis and myelin proteins (Campagnoni and Macklin, 1988; Dowhan, 2009). The importance of strictly regulated lipid composition is underscored by a large number of lipid-related genetic diseases. Supplementary Figure 1 of (Harayama and Riezman, 2018) lists 135 genetic defects in lipid metabolism that cause or contribute to human disease.

The process of spontaneous self-organization of lipid molecules into the lipid bilayer in water is largely due to their hydrophobic properties. When lipids are dispersed in water, their hydrophobic tails promote water molecules to form quasi-regular ‘clathrate cages’ around these hydrophobic parts. Depending on the phospholipid head group, six or more water molecules surround a lipid molecule (Chattopadhyay et al., 2021). When lipid molecules come together, water molecules lose their clathrate cage structure and form more disordered water clusters, thereby increasing the total entropy of the system and making the self-organization of the monolayer of lipid molecules a thermodynamically favorable process (Gao et al., 2022). The free energy is further decreased when two lipid monolayers pack tail-to-tail to form a more favorable arrangement with minimal contact with water–a phospholipid bilayer—the basic structure of biomembranes.

#### 5.2.1 Cholesterol

Cholesterol is amphipathic. It has a polar head with only one hydroxyl group and four rings and a hydrophobic hydrocarbon tail that can readily insert into the hydrophobic interior of cell membranes. The four fused hydrocarbon rings in cholesterol have an almost flat rigid structure, and their contact with other lipids and proteins within the membrane leads to a higher packing density. Thus, cholesterol helps to reduce the penetration of water, gases (e.g., oxygen), and small neutral molecules (e.g., glucose) through the membrane (Shinoda, 2016; Olżyńska et al., 2020). The importance of cholesterol for myelin structure and function can be inferred from its relatively high proportion in myelin (40%) compared to typical cell membranes (25%). A study of electron paramagnetic resonance signals found that cholesterol content strongly influences the membrane’s structural organization and permeability (Subczynski, et al., 2017). High cholesterol content (30%–50%) ensures the high hydrophobicity of the membrane and increases membrane packing. Cholesterol is also a key determinant of membrane fluidity. The critical significance of cholesterol in myelin membrane is further highlighted by a study of mice, which lacked the ability to synthesize cholesterol, and had markedly reduced myelination (Saher et al., 2005). Conversely, the process of myelin repair–remyelination - is more efficient when the rate of cholesterol synthesis is increased (Berghoff et al., 2021).

#### 5.2.2 Phospholipids

Two of the major classes of membrane phospholipids—sphingomyelins and phosphatidylcholines—constitute more than 50% of membrane phospholipids. The long lengths of the hydrophobic tails of these phospholipids—ranging from 14 to 24 carbon atoms—increase the interaction between tails, promote tight packing, decrease the fluidity of lipid association and provide a less permeable barrier for ions allowing for better insulation of axons (Chrast et al., 2011; Montani, 2021).

#### 5.2.3 Glycolipids

Two of the most abundant glycolipids in the myelin membrane are galactocerebroside (GalC) and galactosulfatide (sGalC). Glocolipids’ long alkyl chains are closely aligned–they can form up to eight intermolecular hydrogen bonds. Glycolipids also interact with phospholipids and cholesterol to promote the formation of dense packing in the bilayer of the myelin membrane (Stoffel and Bosio, 1997). Phospholipids and glycolipids are asymmetrically arranged on the membrane, with phospholipids predominating on the inner sheet of the lipid bilayer and glycolipids on the outer sheet (Stoffel and Bosio, 1997). The network of hydrogen contacts among lipids is conducive to the formation of micro lipid rafts, a kind of liquid crystal structures. These densely packed regions decrease the overall motion of the membrane and make it more rigid and more resistant to fluid/solid phase transition, resulting in the phase transition temperature of the myelin membrane above the physiological body temperature. The deficiency of glycolipid molecules impairs the packing of the lipid bilayer, increases membrane permeability, and causes the breakdown of the conductance of myelinated axons. The important contribution of glycolipids to myelin explains the twofold increase in the proportion of glycolipids in myelin compared to typical biomembrane.

### 5.3 Myelin proteins

Myelin in the CNS and the PNS contains a relatively small quantity of proteins, but they constitute a highly diverse group (Jahn et al., 2020). A search for human myelin proteins in UniProtKB yields 223 results (https://www.uniprot.org/, accessed 12/14/2022). These proteins have very diverse sequences, functions, and structures yet share some common characteristics: they are typically small, usually no more than 30 KDa in weight, have long half-lives (Toyama et al., 2013), and are multifunctional. Another feature common to many myelin proteins is that they are either intrinsically disordered proteins (IDP) or have intrinsically disordered regions (IDR) (Dyson and Wright 2005; Raasakka and Kursula, 2020). The absence of a fixed, ordered three-dimensional structure in part or the whole of myelin protein is due to a relatively small proportion of hydrophobic amino acids and a higher proportion of disorder-promoting amino acids - R, K, E, P, and S, which prevent the formation of an ordered structural domain with a stable hydrophobic core (Romero et al., 2001; He et al., 2009). The high conformational flexibility of IDR allows myelin proteins to adopt variable structures depending on their neighboring contacts. Upon binding with other molecules within myelin, IDRs often undergo a disorder-to-order transition known as coupled folding and binding (Wright and Dyson, 2009). IDRs within myelin proteins play an important role in forming multilayer myelin membranes. For example, the disordered region of the myelin protein zero (P0) participates in developing the mature myelin membrane (Raasakka and Kursula, 2020). In the following sections, we will discuss three structurally important and common myelin proteins: proteolipid protein (PLP), myelin basic protein (MBP), and myelin protein zero. These three proteins are representative of the diversity of myelin-associated proteins and are illustrative of some of the key features of this protein group.

#### 5.3.1 Proteolipid protein (PLP)

PLP is the most abundant myelin protein in the CNS, where it constitutes 38% of the total myelin protein mass. In contrast, the amount of PLP in the PNS is minimal (Jann et al., 2020). PLP1 gene encodes human PLP and is expressed in oligodendrocytes, but also in oligodendrocytes, astrocytes, and even in some neuronal progenitor cells (Harlow et al., 2014). A high level of PLP in myelin is required to preserve myelin integrity. The key role of PLP in the formation of a compact multilayer membrane structure is to bring myelin membranes closer to each other. A reduction in PLP content by 50% causes altered myelin ultrastructure and axonal pathology (Lüders et al., 2019). Mutations in PLP1 gene may result in hypomyelination and a spectrum of neurogenetic disorders, including Pelizaeus-Merzbacher disease and spastic paraplegia 2 (Inoue, 2019; Wolf et al., 2019).

PLP is a highly conserved hydrophobic protein. It comprises four transmembrane segments spanning residues 10–36, 64–88, 152–177, and 234–260, of which 79 amino acids (76%) have hydrophobic side chains. Both the N- and C-termini of PLP are on the cytoplasmic side. PLP exists as two isoforms (UniProt P60201). The larger isoform weights 30 kDa and is 277 amino acids long, and the shorter isoform, PLP/DM20, is 26 kDa and is identical in sequence to the longer version, except for a deletion of 35 amino acids in the intracellular loop (Spörkel et al., 2002). A recent publication shows that both full-length human PLP and its shorter DM20 isoform have a dimeric, α-helical conformation and discusses structural differences between the isoforms in terms of their impact on protein function and interaction with lipids (Ruskamo et al., 2022).

Experimental 3D structural information for the full-length PLP or DM20 has not been reported, but there is X-ray data of a small fragment of the PLP chain (Uniprot P60201-1: residues 45–53) in the loop between the first and second transmembrane helices (PDB structure 2XPG). This peptide (KLIETYFSK), which covered only 3% of the PLP molecule, forms a complex with HLA class I histocompatibility molecule HLA-A*0301 (McMahon et al., 2011) and may therefore play a role in autoimmunity. It is interesting to note in this context that patients with multiple sclerosis, a chronic demyelinating disorder of CNS, exhibit elevated T-cell and antibody responses to PLP (Greer et al., 2020).

The three-dimensional structure of the PLP was recently predicted using the highly-accurate AlphaFold method (Jumper et al., 2021). AlphaFold predicted that the largest part of the PLP chain forms helical structures (https://alphafold.ebi.ac.uk/entry/P60201). In the predicted model, most residues (with the exception of residues 110–140) have relatively small expected position errors.

#### 5.3.2 Myelin basic protein (MBP)

MBP is the second most abundant myelin protein in CNS: it constitutes about 30% of dry protein mass in CNS myelin. MBP is less abundant in the PNS, where it accounts for only 5%–18% of the total myelin protein (Garbay et al., 2000). MBP has a number of different functions: it interacts with other proteins and participates in the transmission of the extracellular signal to the cytoskeleton and tight junctions (Boggs et al., 2006). MBP was called the ‘executive’ molecule of the myelin membrane in view of its critical role in compact myelin sheath formation (Moscarello et al., 1997).

In mammals, the MBP gene that codes for MBP comprises seven exons. Differential splicing of the primary mRNA leads to different isoforms of the protein. Not all of them are involved in axon myelination: for example, isoform 1 (UniProt P02686-1, 304 amino acids, 33.1 kDa) participates in the early brain development before the onset of myelination (Vassall et al., 2015). The so-called ‘classic myelin isoforms’ are part of the myelin membrane mostly; they vary in their molecular mass from 14 to 21.5 kDa. The 18.5-kDa isoform (UniProt P02686-5; 171 amino acids) is the most abundant isoform of MBP in mature human myelin in the CNS, while the 17.2 kDa isoform (UniProt P02686-6; 160 amino acids) is the major MBP isoform in the PNS.

In addition to isoform variability, MBP isoforms undergo a large number of post-translational modifications, which include phosphorylation, citrullination of arginyl residues, acetylation of lysine, and other reactions (Zhang, 2012). Such post-translational modification gives rise to eight charged isomers (C1-C8) of isoform 18.5 kDa. The mostly unmodified C1 isomer has the highest positive charge (net charge of +19 at pH 7). In contrast, the mostly modified isomer C8 has the smallest net positive charge of all the isomers (net charge of +13 at pH 7) because of deimination (citrullination) of six arginine residues into uncharged non-canonical amino acid citrulline at positions 26, 32,123, 131, 160, 170 (UniProt P02686-5) (Wood and Moscarello, 1989; Tranquill et al., 2000). The irreversible citrullination reaction reduces the positive surface charge of the MBP, thereby weakening the interactions of the MBP with negatively charged lipids, which leads to a decrease in myelin stability (Martinsen and Kursula, 2022). The process of citrullination may also have clinical implications. In one fulminant case of multiple sclerosis, known as ‘Marburg variant,’ deimination of 18 of 19 arginyl residues to citrulline within acutely demyelinating plaque led to a dramatic decrease in MBP positive charge. Such a decrease of positivity in MBP is incompatible with MBP function in compacting myelin and may have triggered fatal autoimmune demyelination in this patient (Wood et al., 1996). In this context, it is notable that multiple sclerosis patients’ T cells appear to preferentially respond to citrullinated MBP, which suggests that citrullination of MBP may be involved in the induction or perpetuation of multiple sclerosis (Tranquill et al., 2000).

Different charge isomers may have different functions in various stages of myelin development. The most positive charged variants C1, C2, and C3 are part of a stable myelin sheath, while C8 charge variant might be of importance during the sheath’s development (Moscarello et al., 1994). C1 isomer of isoform 18.5-kDa is characterized by low hydrophobic content - about 25% of all its residues are hydrophobic (Harauz and Boggs, 2013). This is consistent with the localization of this MBP isoform to the cytoplasmic part of the myelin membranes (Figure 1B). The role of MBP is to bring closer together two apposing negatively charged cytoplasmic leaflets of the myelin membrane that form the major dense line. Force–distance measurements show that maximum adhesion force and minimum cytoplasmic spacing occur when each negative lipid in the membrane can be bound to a positively charged lysine or arginine group on MBP (Min et al., 2009). Excess of MBP causes the formation of a weak gel between myelin surfaces, while an excess of negative charge causes electrostatic swelling of the water gap (Smith, 1992). Thus, excess or deficiency of MBP causes the myelin bilayers to repel each other and may lead to the destruction of myelin (demyelination).

The lipid composition of the myelin leaflet has a major impact on its interactions with MBP. A cholesterol content of 44% in myelin yields the most thermodynamically favorable MBP interaction and is optimal for membrane compaction and thermodynamic stability (Träger et al., 2020). In addition to its structural importance–*via* interactions with lipids and myelin membrane-associated proteins–MBP also interacts with a large group of proteins related to protein expression and may play a regulatory role in myelinogenesis (Smirnova et al., 2021).

#### 5.3.3 Myelin protein zero

Myelin protein zero molecule (P0) is expressed in higher vertebrates only in the PNS (Yoshida and Colman, 1996), where it makes up more than 50% of all myelin protein. P0 synthesis is regulated by Schwann cell/axon interactions, the so-called ‘axonal signal’. Axons can up- and downregulate the expression of Schwann cell genes *via* a cyclic adenosine monophosphate (cAMP)—dependent pathway (Lemke and Chao, 1988).

**FIGURE 2:**
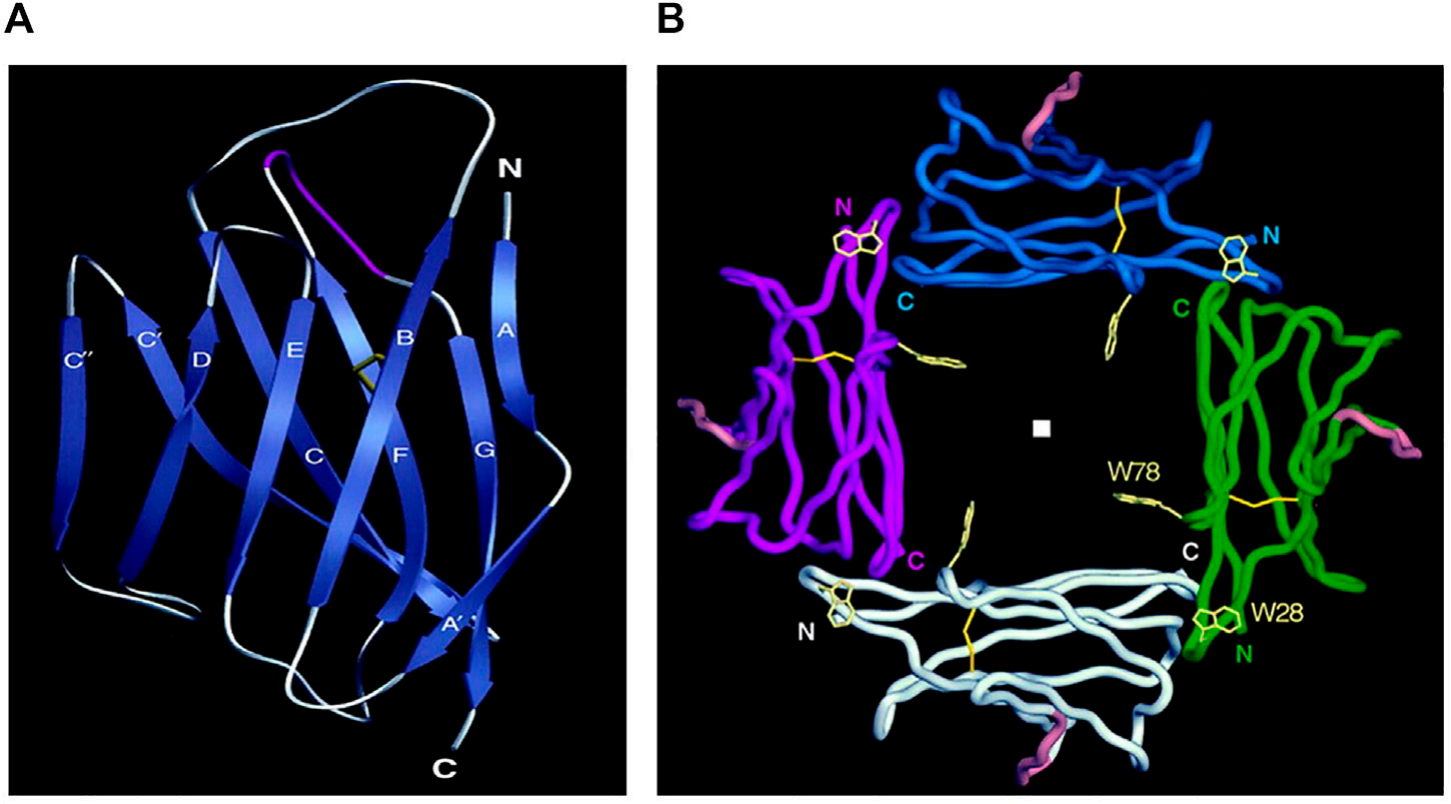
Structure of myelin protein zero. **(A)**: Extracellular domain with labeled 10 beta strands in the standard immunoglobulin designation. Strands A (2-4), B (17-24), D (70-73) and E (82-85) make up one beta-sheet, and strands A' (8-11), C (33-40), C' (47-54), C" (57-60), F (93-101) and G (109-118) make up the other beta-sheet. Five residues make major contributions to the formation of the dimer: Trp 57, Asp 61 (C—C' loop), Lys 84, Gly 85 (loop C'—C"), and Ser 106 (loop C"—D). **(B)**: Four extracellular Ig-like domains form a tetramer The position of the fourfold axis is indicated by a square. N- termini and C termini point the beginning and the end of amino acid chains, respectively. Trp-28 makes van der Waals contacts with main chain atoms of the opposing B–C loop Loop. Adapted from Shapiro L et al., Neuron. Sep;17(3):435-449.

The human P0 molecule (P25189 ·MYP0_HUMAN) is 248 amino acids long and consists of an N-terminal region (29 residues) and three domains. The structure of two rat and human P0 extracellular domains have been determined with high resolution by X-ray crystallography (rat—PDB ID 1NEU; Shapiro et al., 1996; human—PDB ID 3OAI; Liu et al., 2012). The structure of the extracellular domain (125 residues) is similar to typical variable domains of immunoglobulins with two beta sheets–sandwich-like structure with a set of Ig-conservative residues, including a pair of Cys residues in the B- and F—strands that form a disulfide bond contact between the two sheets, and Trp residue in the C-strand, which is involved in many intradomain contacts (Figure 2A). An important consequence of the homophilic adhesion properties of extracellular domains of P0 molecule is their ability to form dimers and tetramers. Two extracellular P0 domains form antiparallel dimers, and two neighboring dimers create a tetramer between lipid membranes (Figure 2B). The dimer and tetramer formation between extracellular domains is strengthened through the participation of the two other P0 domains (Shapiro et., 1996; Plotkowski et al., 2007).

The 27 residues-long transmembrane domain of P0 forms a single helix. The role of this domain in the formation of P0 dimers and tetramers was analyzed in detail byPlotkowsky et al., 2007. An important feature of the transmembrane domain is the presence of a conserved glycine zipper motif–GxxxGxxxG (in human 159GAVIGGVLG167), which is conserved across many membranes’ protein sequences. Zipper motif is the primary packing interface of the transmembrane helix. The interaction between helices within the membrane determines the correct orientation Ig domains for dimer formation in extracellular space.

The third domain of P0, the 67 residue-long C-terminal cytoplasmic domain, plays a role in tetramer formation. This domain exists in a disordered state, typical for many membrane proteins that interact with lipids, and has a high content of positive charged R, K, and H residues. In the sequence of the human domain shown below, these residues are bolded and marked in red.

R YCWL RR QAALQ RR LSAME K G K L HK PG K DAS KR G

R QTPVLYAMLD H S R ST K AVSE KK A K GLGES RK D KK

Thus, there are 23 positive charged residues in the third domain that are approximately evenly distributed throughout the sequence and only six negative charged residues. Electrostatic interactions of mostly positive cytoplasmic domain with the negative cytoplasmic phospholipid headgroups are largely responsible for the formation of a stable helical-ordered protein structure (Raasakka and Kursula, 2020). As a result of these interactions, important structural transformations occur within myelin, which brings two neighboring P0 molecules together and ‘tighten’ the two adjacent membranes. These contacts have a similar function to contacts between MBP within the cytoplasmic part of the myelin membranes, considered above. Four neighboring P0 extracellular domains are assembled as a tetramer with a fourfold symmetry axis Figure 2B. Because this tetrameric association is so stable, it may be considered the main structural unit of the native myelin membrane structure in PNS (Thompson et al., 2002).

## 6 Myelin: History of discovery and questions for future research

Van Leeuwenhoek was the first to detect myelinated fibers in 1717, and Rudolf Virchow described myelin’s chemical nature in 1854 and gave it its name. More than another century passed until it was conclusively established that CNS myelin is formed by oligodendrocytes (Bunge et al., 1962). In 1878, Ranvier established that myelin coverage of axons is not continuous but periodically interrupted by non-myelinated sections, which we now call the ‘nodes of Ranvier’ (NR). Only very recently, the molecular mechanism of the NR assembly was described in detail (Rasband and Peles, 2021), yet many unresolved questions remain. For example, it is not known how the distance between NR is regulated during the process of myelination. The distance between the nodes changes in accordance with the growth of the axon, but how this information is conveyed to oligodendrocytes is unknown. The rich and fascinating history of myelin research from the Renaissance to the present was a subject of a recent review (Boullerne, 2016).

Traditionally, the study of myelin has focused on understanding its properties as an axonal insulator. The current trend in the field is to enlarge the focus to encompass the entire complex involving myelin, oligodendrocyte, axon, and other cells involved in myelination. From this perspective, the study of myelin is not so much an investigation into its complex chemical nature but of the interrelationship and interdependence between living cellular elements that contribute to myelination (Bonetto et al., 2021). This perspective allows one to appreciate the system’s plasticity–how functional and structural changes occur in response to changes in the living organism. An example of how this shift in focus yields new insights into myelin biology is the newly described concept of ‘adaptive myelination’ (Bechler et al., 2018; Bloom et al., 2022). It is clear that the investigation will not end at this stage of cellular plasticity but will proceed to the next level of organizational complexity: the neuroplasticity of the organ level–that of the brain and nervous tissue.
